# MCT8 deficiency in a patient with a novel frameshift variant in the *SLC16A2* gene

**DOI:** 10.1038/s41439-021-00142-0

**Published:** 2021-02-16

**Authors:** Kei Wakabayashi, Hitoshi Osaka, Karin Kojima, Taichi Imaizumi, Toshiyuki Yamamoto, Takanori Yamagata

**Affiliations:** 1 Department of Pediatrics, Jichi Medical University, Tochigi, Japan; 2 Institute of Medical Genetics, Tokyo Women’s Medical University, Tokyo, Japan; 3 Department of Pediatrics, St. Marianna University School of Medicine, Kawasaki, Japan

**Keywords:** Genetic predisposition to disease, Paediatric neurological disorders

## Abstract

MCT8 deficiency is an X-linked recessive disorder. We report the case of a 2-year-old Japanese boy with MCT8 deficiency caused by a novel frameshift variant, NM_006517.5(SLC16A2_v001):c.966dup [p.(Ile323Hisfs*57)]. He presented no head control and spoke no meaningful words, indicating severe developmental delay. Although missense or in-frame mutations of *SLC16A2* are usually related to milder phenotypes and later-onset pyramidal signs, loss-of-function mutations are expected to cause severe clinical symptoms.

Monocarboxylate transporter 8 (MCT8), which belongs to the solute carrier family (SLC), consists of 12 transmembrane domains and is encoded by *SLC16A2* on Xq13.2^1^. MCT8 deficiency, also known as Allan–Herndon–Dudley syndrome, is an X-linked recessive disorder that presents with intellectual disability, motor retardation, spastic quadriplegia, and hypotonia from infancy due to the impaired uptake of 3,3’,5-triiodothyronine (T3) into neurons^2–5^ The prevalence of MCT8 deficiency is unknown. To date, over 160 affected individuals and over 100 mutations in the SLC16A2 gene have been reported in patients with MCT8 deficiency, including missense, nonsense, deletion, insertion, frameshift, and splice site mutations^3,6^. We herein report a case involving a patient with MCT8 deficiency with a novel frameshift variant of *SLC16A2* who presented with dystonia and cerebral hemisphere atrophy from early infancy.

The patient was a 2-year-old Japanese boy born to nonconsanguineous parents with no family history of neurological or endocrine disorders. He was born at 37 weeks of gestation by cesarean section without neonatal asphyxia. At birth, his weight was 2575 g (−0.85 SD) and his body length was 47.1 cm (−0.38 SD). Newborn screening by tandem mass spectrometry revealed no abnormalities, and the automated auditory brainstem response was normal. He was discharged from the maternity clinic at 6 days of age with no special remarks on his perinatal history. No physical or neurological abnormalities were found at 1 month. At 6 months of age, he was referred to our hospital with a lack of head control. At consultation, his weight was 7425 g (−0.62 SD) and his body length was 68.0 cm (+0.14 SD). He never achieved head control or the ability to roll over and had bilateral dystonia of the upper limbs. He was hypotonic, with increased muscle extensibility and passivity. The slip-through sign was positive. Deep tendon reflexes, including the biceps, triceps, brachioradialis, patella, and Achilles reflexes, were normal. Babinski and Chaddock reflexes were positive. Traction response revealed head lag. Primitive reflexes were positive for the palm grasp reflex, plantar grasp reflex, and asymmetrical tonic neck reflex. The first phase of the Moro reflex was observed. Furthermore, he presented with umbilical hernia and bilateral hydrocele testes. He had no facial abnormalities. Brain magnetic resonance imaging (MRI) at 6 months of age showed markedly delayed myelination and cerebral atrophy (Fig. 1a, b). A laboratory analysis showed abnormal thyroid function, with a normal thyroid-stimulating hormone (TSH) level of 2.92 µlU/ml (normal range: 0.62–4.90), a high free T3 level of 7.14 pg/ml (normal range: 2.91–4.70), and a low free T4 level of 0.73 ng/ml (normal range: 1.12–1.67). We suspected MCT8 deficiency because of the combined presentation of delayed myelination on brain MRI and thyroid dysfunction with a high free T3 level and a low free T4 level pattern. After obtaining written informed consent, blood samples were obtained from the family for a molecular diagnosis. PCR-based direct Sanger sequencing of *SLC16A2* identified a novel 1-bp duplication, NM_006517.5(SLC16A2_v001):c.966dup in exon 3, leading to a frameshift, p.(Ile323Hisfs*57) (Fig. 1c). The patient’s mother was a carrier of the mutation. Currently, the patient’s weight is 11.4 kg (−0.34 SD) and his height is 86.3 cm (+0.03 SD) at 2 years and 1 month of age. He shows severe dystonia and developmental delay, without head control or the ability to turn over or speak meaningful words.

**Fig. 1:**
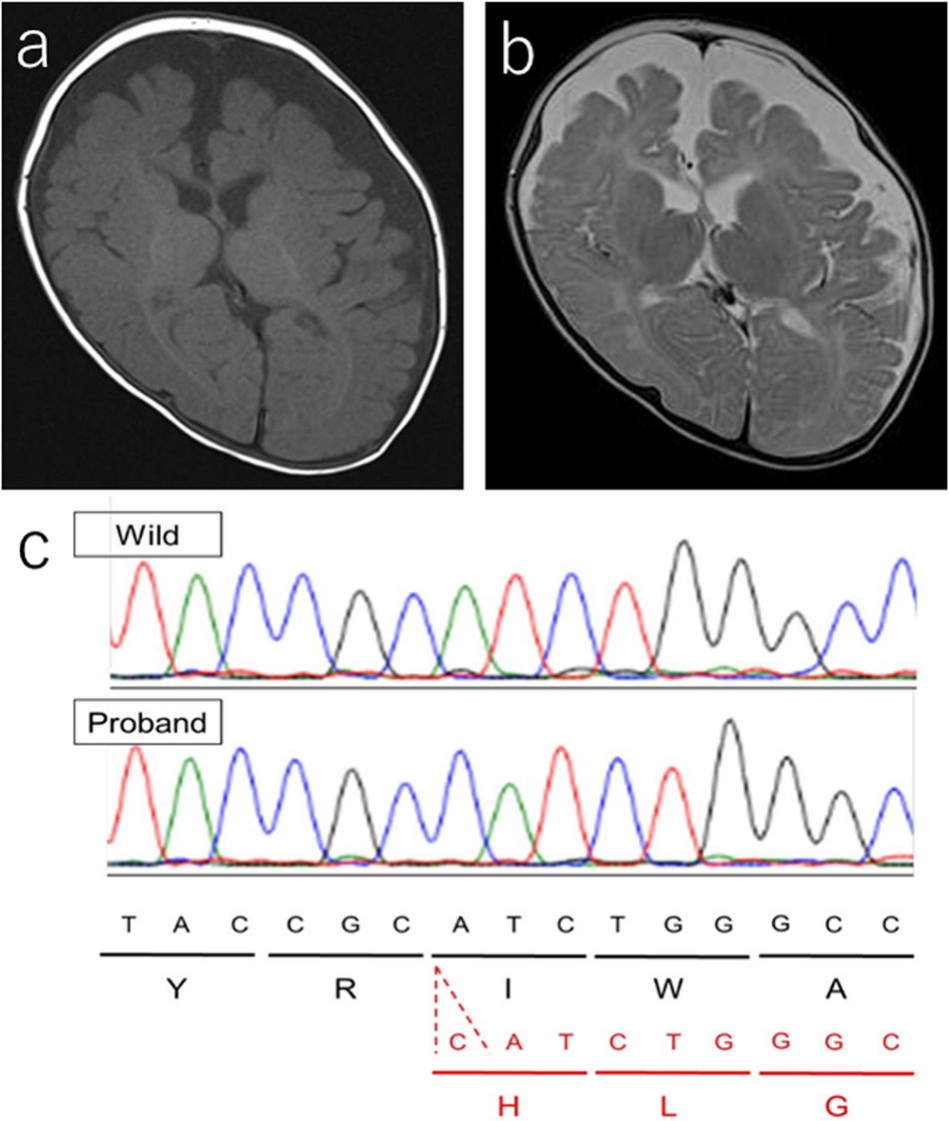
The clinical and genetic presentation of this case. Brain magnetic resonance imaging of this patient at 6 months of age (**a** T1-weighted axial image, **b** T2-weighted axial image). The anterior and posterior limbs of the internal capsule and corona radiata show a high signal intensity. On the other hand, the low signal intensity in the frontal white matter suggests delayed myelination. The expansion of the subarachnoid space indicates cerebral hemisphere atrophy. **c** An electropherogram showing the mutation in this case. One nucleotide duplication, c.966dup [p.(Ile323Hisfs*57)], was found in *SLC16A2* exon 3.

Patients with MCT8 deficiency typically show hypotonia and developmental delay in early infancy, with delayed myelination. In later childhood, pyramidal and extrapyramidal signs appear^6^. Our case showed a severe phenotype, with both dystonia and cerebral atrophy starting from early infancy. As far as we know, five cases of MCT8 deficiency due to a frameshift variant have been reported (Table 1)^7–11^. In all cases with frameshift mutations, the patients were unable to sit without support or to speak meaningful words, which was consistent with the severe symptoms of our case. Including the present case, the high rate of cerebral hemisphere atrophy (4/5) also indicates that frameshift mutations are associated with severe central nervous system damage in comparison to overall MCT8 deficiency, where the rate of cerebral atrophy was reported to be 17–41.6% in all cases^6^. MCT8 deficiency is predicted to be caused by the loss of MCT8 function^6^. Some patients with missense or in-frame mutations show a mild phenotype, with the ability to sit up, walk, and speak^6^. An in vitro analysis revealed that the MCT8 found in these mild phenotypes was expressed on the cell membrane and retained its ability to take up T3, while frameshift mutations prevented the expression of MCT8^1^. In contrast, the present case and all cases with frameshift mutations showed a severe phenotype (Table 1). Both protein instability and nonsense-mediated mRNA decay may account for the profound loss of function.

**Table.1:** Comparison of genotypes and clinical symptoms in this case and previous study with frameshift variant.

MCT8 mutation	c.966dup (this case)	c.735_760dup	c.1343_1344insGCCC	c.1063_1064insCTACC	c.1649delA	c.1614dupC
Age, years	2	2	5	21	4	1
Dystonia	+	+	+	+	+	NA
Cerebral hemisphere atrophy	+	+	+	+	+	NA
Delayed myelination	+	+	+	+	+	+
Sitting without support	−	−	−	NA	−	−
Independent gait	−	−	−	NA	NA	−
Speaking meaningful words	−	−	−	NA	−	−

*NA* not available.

In this case, abnormal thyroid function—a characteristic of MCT8 deficiency—was found at diagnosis (elevated free T3 level, decreased to normal free T4 level, and normal to elevated TSH level)^12^. However, thyroid function in this disease may show some normal values^6^. If dystonia and cerebral hemisphere atrophy are noted, MCT8 deficiency should be suspected, and thyroid hormone measurements should be repeated.

In conclusion, we encountered a case of MCT8 deficiency with a novel frameshift variant, c.966dup [p.(Ile323Hisfs*57)]. A loss of MCT8 function due to a frameshift variant is expected to result in severe clinical symptoms.

## Data Availability

The relevant data from this Data Report are hosted at the Human Genome Variation Database at 10.6084/m9.figshare.hgv.2972.
